# Spatial coding of conspecifics in the electrosensory system: Early segregation of information streams

**DOI:** 10.1371/journal.pone.0348018

**Published:** 2026-05-12

**Authors:** Oak E. Milam, Gary Marsat

**Affiliations:** Department of Biology, West Virginia University, Morgantown, United States of America; Nathan S Kline Institute, UNITED STATES OF AMERICA

## Abstract

Localizing the source of a signal requires sophisticated neural mechanisms, and we are still uncovering the coding principles that support accurate spatial processing. Weakly electric fish can detect and localize distant conspecifics, but the way this spatial information is encoded is unclear. Here, we investigate the spatial representation of conspecific signals in the hindbrain to determine how the properties of the heterogeneous population of pyramidal cells affect the spatial coding accuracy of conspecific signals. We hypothesize that specific subsets of cells provide more accurate spatial information about conspecific location. We stimulated the fish with an artificial signal that replicates both the spatial and temporal structure of conspecific signals. We recorded from cells with various receptive field positions covering the entire body surface and analyzed the spike train with spike-train distance metrics to determine how accurately the location of the stimulus is encoded. We found that some pyramidal cells (such as ON-type and those within the deep layer) encode the spatial information more accurately, while other subgroups (OFF-type and superficial layer) provide less accurate information. Our results help us understand how conspecific location is represented across the heterogeneous population of cells and suggest that segregation of the spatial information stream begins early in the sensory pathway.

## Introduction

Efficient coding of sensory signals is a key principle in neuroscience [1–4], and this includes encoding their spatial aspect to guide targeted behaviors, such as approaching a mate or avoiding a foe [1,5]. Yet, how populations of neurons represent this spatial information to optimize accuracy remains a topic of debate. We investigate how spatial information is encoded in the hindbrain of weakly electric fish to characterize how neural heterogeneity is shaped to encode the signal accurately.

Brown Ghost Knifefish generate electric signals to communicate and guide movements during social interactions. The continuous, quasi-sinusoidal electric organ discharge (EOD) is generated via an electric organ located in the tail [2]. The EOD drives the baseline discharge of tuberous electroreceptors (P-units) distributed across the entire body. The afferents from each P-unit provide trifurcated, unilateral input to different subtypes of pyramidal cells located in the three topographic maps of the electrosensory lateral line lobe (ELL): the lateral segment (LS), the centro-lateral segment (CLS), and the centro-medial segment (CMS; for review see [3,4]). Multiple topographic maps in the ELL are comprised of a heterogeneous network of ON and OFF-type pyramidal cells [6–8]. Pyramidal cells are organized in a columnar layout, containing superficial, intermediate, and deep-type pyramidal cells. Within each map, pyramidal cells exhibit varying response properties and center-surround receptive field parameters [3,6,8,9]. Receptive fields in the LS map are the largest, the CMS map contains the smallest receptive fields, and receptive fields in the CLS map are intermediate. Theoretical studies show that smaller receptive fields generally support more accurate spatial representation of spatially localized signals such as small prey [10,11]. The response properties of ELL neurons also suggest that different neural maps are specialized for certain behavioral tasks [12–15]. Localized prey-like signals are particularly well represented in CMS [11] while CLS and LS segments accurately encode conspecific signals and are involved in communication behaviors [14–16]. For these reasons, our study will focus on the CLS and LS segments. Despite our understanding of map differences and characterization of temporal response properties across sub-populations of pyramidal cells, little is known about how the spatial aspect of conspecific stimuli is represented across these neurons. Since the ELL constitutes a bottleneck for the electrosensory information being relayed to higher brain areas, the neural properties of its heterogeneous neural population will dictate sensory acuity; the ELL is therefore an ideal target for examining sensory coding strategies.

Recent field and lab studies on interacting weakly electric fish indicate that these animals possess an aptitude for detecting and localizing conspecific signals in their environment, even in conditions where sensory cues are limited [17–23]. The diffuse nature of these signals (affecting peripheral receptors covering the entire body, i.e., “global signal”) suggests that the central nervous system must discriminate between small differences in the spatial pattern of the signal to encode conspecific location accurately. Although behavioral observations clearly demonstrate their sensory capacity, the mechanism by which the nervous system accomplishes this task remains unknown. Our goal in this study is to clarify how the primary electrosensory area of the nervous system encodes the location of a conspecific. We hypothesize that the encoding of spatial information involves a heterogeneous neural representation across pyramidal cell subtypes.

Weakly electric fish provide a tractable system for examining population-level encoding, and similar principles of efficient population coding have been explored in other modalities and species. Studies in auditory and visual systems, for instance, have demonstrated that neural populations achieve optimal encoding by distributing noise and tuning properties across neurons to maximize mutual information under constraints [24–26]. Such models predict a specialization in subpopulations—each contributing uniquely depending on response precision, receptive field structure, or the nature of the stimulus feature being encoded [27–29]. These ideas resonate with our hypothesis that the heterogeneity of ELL pyramidal cells in weakly electric fish supports a distributed population code for conspecific signals.

## Materials and methods

### Animals

Wild-caught *Apteronotus leptorhynchus* of unknown sex and age were obtained from commercial fish suppliers. The tanks’ water conductivity was maintained at 200–300 µS/cm and temperatures of 26–27°C. The West Virginia University’s Institutional Animal Care and Use Committee IACUC approved all procedures (protocol #1811019244).

### Electrophysiology

Surgical techniques follow previously established procedures [30,31]. Briefly, a fish was anesthetized with tricaine methanesulfonate (Western Chemical, Inc.) and respirated during surgery. A local anesthetic (Lidocaine HCL 2%, Hospira, Inc.) was applied, and the skin overlying the craniotomy site was removed. A fixed bar with a circular opening was glued to a portion of the exposed skull for stability. The fish was immobilized with an injection of tubocurarine chloride pentahydrate (0.2 mg ml^-1^, TCl inc.). The experimental tank contained water with a conductivity of 250 (±10) µS/cm and a temperature of 26 (±1) °C. The portion of the skull above the ELL was removed. A cone was secured to the fixed post, allowing top-down access to the exposed ELL. Melted resin was used to form a watertight seal between the ventral opening of the cone and the skull around the exposed ELL. ACSF was applied to the brain. This cone allows for full-body submersion into the experimental tank during recordings, while preventing the brain from coming into contact with the tank water. *In vivo*, single-unit recordings of the lateral segment (LS) and centrolateral segment (CLS) were performed using metal-filled extracellular electrodes [32]. The fish was maintained under local anesthesia throughout the recording period. Recordings were amplified (A-M Systems, Model 1700) and data recorded (Axon Digidata 1500 and Axoscope software, Molecular Devices) at a 20kHz sampling rate. Pyramidal cells of the LS and CLS were identified based on the blood vessel landmarks, depth of penetration (in the dorsal-ventral plane), and response properties of the neurons [33,34]. General properties of the cells were determined using global electrodes (see next section).

The receptive field center of each neuron was delineated experimentally by moving a local dipole across the body surface and determining the edge of the classical receptive field where the neurons responded with their characteristic ON-center or OFF-center responses. To do so, the small dipole was coarsely moved across the surface of the fish to roughly identify the receptive field extent. The dipole was then moved in small increments around the edges of the receptive field to identify positions where the response (visualized with a PSTH) did and did not show peaks and through relative to the stimulus period (5 Hz sinusoidal AM with 20% contrast). The coordinates of the corresponding electrode positions were recorded as a relative value (0–1) along the rostro-caudal and dorso-ventral axes.

### Stimulation

All stimuli were sampled at 20 kHz and created in MATLAB (MathWorks, Inc.). Our stimulation procedure replicates the amplitude modulations (AM) experienced during social interactions. The baseline EOD was recorded between the head and tail of the fish. Each EOD cycle triggered a sine wave generator (Rigol DG1022A) to output one cycle of a sinusoidal signal with a matching frequency to the fish’s EOD. This signal was then multiplied using a custom-built signal multiplier by the AM stimulus to create the desired modulation of the electric field. Stimuli were played through a custom made stimulus isolator into the experimental tank using one of three configurations: a global stimulation, via two 30.5 cm carbon electrodes arranged parallel to the longitudinal axis of the fish; a local stimulation, via two silver chloridized electrodes 0.5 cm apart positioned at various positions near the skin surface; an artificial conspecific stimulation (i.e., “fishpole”), via two silver chloridized electrodes embedded in agarose with a conductivity of 35 µS [35–37]. Stimulations for global and local configurations were adjusted to provide ~20% contrast relative to the baseline EOD strength.

**Fig 1:**
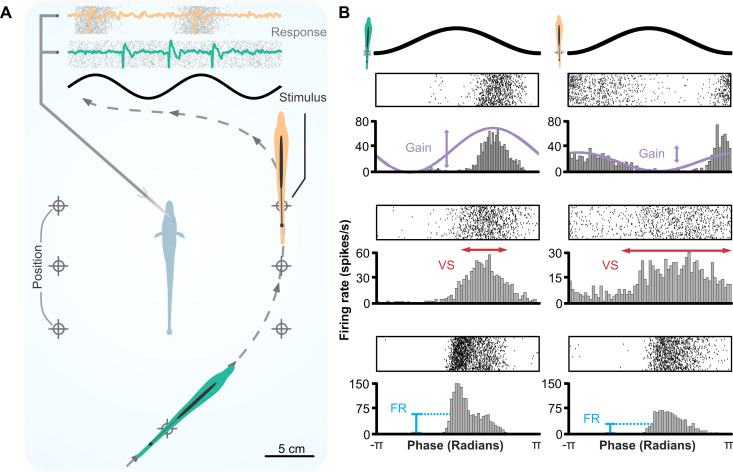
Spatially realistic conspecific signals and response strengths in the ELL. **(A)** Schematic of the experimental design. An immobilized *A. leptorhynchus* (center, light blue) is stimulated using a conspecific dipole mimic (i.e., “fishpole”) positioned at various locations around the experimental tank (at distances of 10 cm), while recording extracellularly from ELL pyramidal neurons in vivo. Neural responses to two cycles of the conspecific stimulus (AM shown in black, top) are shown as raster plots (grey, top) overlayed with a trace of the raw neural recordings. The examples highlight differences in the pattern of the spike train responses for encoding spatial stimuli (orange and green). **(B)** Measurement of response strength to stimulations with conspecific stimulus from different stimulation sites (orange site for **A.**: left column; green site: right column). Raster plots of the responses are shown above, and the corresponding peristimulus time histograms (PSTH) are shown below. Changes in the spatial location and orientation of the conspecific stimulus can elicit increases or decreases in the spike train response of ELL pyramidal cells. Responses to the spatial stimulus vary in stimulus-response gain (top, purple), vector strength (middle, red), and mean firing rate (bottom, blue). For each of these three measures, we chose a different cell in order to illustrate most clearly differences in the measure exemplified.

The fishpole signal was calibrated to match the amplitude of the electric field potential of real fish recorded from 24 different positions and distances around the fish (n = 7 fish, of mixed EOD frequency and size). Specifically, the geometry and voltage applied to the dipole were adjusted until the mean amplitudes measured at each of the 24 sites matched the means from real fish. The resulting dipole had one of the silver wires representing the rostral end of the fishpole (5.5 cm long), and the other silver wire representing the caudal end (0.1 cm long). The two wires were separated by 4 cm, yielding a fishpole with 9.6 cm in length and a zero-plane potential located at ~70% of the rostral to caudal body length, in accordance with previous observations [38,39]. A solidified agarose body was carved to match the body shape of a real fish. The fishpole was positioned in the experimental tank at three different orientations/azimuths (0, 45, 90°), and seven different locations around the fish, 10 cm away. The 7 locations were the operculum, mid-body, and the zero-potential plane for both ipsilateral and contralateral sides of the fish (6 locations), and 1 location directly caudal to the fish (see Fig 1A). We use the term, orthogonal, to describe stimulus positions where the rostral end of the fishpole is oriented toward the receiving fish. Sinusoidal AM (SAM) stimuli were 40 s long, modulated at 30 Hz, and were played through the fishpole during the experiments.

### Data analysis

All analyses described here were performed using MATLAB. Spike trains collected from experimental recordings were first binarized into a sequence of zeros (no spike) and ones (spike) sampled at 2 kHz. The binarized sequence was transformed into instantaneous firing rates by convolving it with a Gaussian filter. The s.d. of the Gaussian curve was 5 ms for our main 30 Hz stimulus -roughly one sixth of the period- to preserve the phase-specific structure while providing a smooth estimate of instantaneous firing rate. We used either the binarized spike train or the instantaneous firing rate (see below) that were separated into 1-second, 50% overlapping segments. Choosing a longer window would allow a better localization accuracy estimate [29,40], so the actual discrimination performance numbers we present serve primarily as a benchmark to compare different sub-populations of neurons. The specific choice, 1s, is somewhat arbitrary but realistic: experiments on perceptual decision in other species have identified the 0.2-2s range as a window over which sensory inputs can integrate and contribute to decision-making [41–43]. Many of these types of experiments use a 1s stimulus to study the neural bases of sensory-based decision. Therefore, we estimate that 1s is a realistic choice for the window over which spatial information could be integrated.

Statistical analyses were performed using the MATLAB statistical analysis toolbox and custom-made scripts. The data were tested for normality and then accordingly analyzed using 2-way (or n-way) ANOVAs unless otherwise noted. Following all ANOVAs, post hoc comparisons were made using a Tukey-Kramer test. Mean differences were considered statistically significant when p < 0.05.

### Gain

The stimulus-response gain (*G*) to SAM stimulation was calculated according to:


$$G(f)=\sum Q\left(f_{i}\right)$$ (1)

where *Q* is the power spectral density of the convolved spike train, *f*_*i*_ are the frequencies within ± 0.5 Hz of the target SAM frequency (30 Hz). A larger stimulus-response gain value indicates a larger response from the neuron to a stimulus at the target frequency.

### Vector Strength

Using the timing of the spikes in the binarized spike train, the strength of phase locking to SAM stimulation was calculated according to:


$$s=\frac{\left(\sum {x_{i}}^{\text{2}})+(\sum {y_{i}}^{\text{2}})\right)}{p}$$ (2)

where *p* is the number of spikes, and *x* and *y* are the sine and cosine phases of the stimulus at which the *i* spike occurs [44,45]. The vector strength, *s*, quantifies the precision and clustering of responses to a given phase of the stimulus cycle, with 0 being equal responses at all phases of the beat, and 1 being a perfectly precise response at a single phase of the beat.

### Discrimination analysis

Our discrimination analysis is based on a weighted Euclidean distance analysis that relates directly to the information carried by a population of neurons to discriminate between stimuli (see [29] for more details). Here, we compare stimuli from different locations and use one of three measurements to quantify response strength: mean firing rate (i.e., mean number of spikes per 1 s window), vector strength, or gain for each 1-second response segment. A weight is assigned to each neuron for each pair of stimuli being compared. The weight is based on the Kullback-Leibler divergence in the response distributions for each stimulus. The weight is normalized to 1 across neurons within a population response, and the response strength is then multiplied by this weight. Population responses are then taken as data points in Euclidean space, where each dimension represents the weighted response of one neuron in the population. An ensemble of population responses is thus considered, each of which is composed of a random 1-second segment of response from a subset of *n* neurons from the population (*n* will be varied, see below). The Euclidean distances between responses to the same stimulus and across different stimuli are then compared. Larger distances indicate less similarity between spike trains of the neuron ensemble. Stimuli that can be easily discriminated will elicit responses that are very different (i.e., large Euclidean distance) relative to the variability across responses to the same stimulus. The weighting procedure allows to optimize the decoding efficiency by assigning a stronger contribution to the Euclidean distance to neurons that carry more information about the difference in the stimuli. The distributions of Euclidean distances for responses to the same stimulus, *P(D*_*xx*_*),* and across the two stimuli being compared, *P(D*_*xy*_*),* are then used in a receiver operating characteristic (ROC) analysis. Receiver operating characteristic (ROC) curves were generated by varying a threshold distance value *T*; for each threshold, the probability of discrimination (*P*_*D*_) is calculated as the sum of *P(D*_*xy*_ > T), and the probability of false discrimination *(P*_*F*_) is calculated as the sum of *P(D*_*xx*_ > T). The error probability is taken as the minimum error, *E,* across thresholds:


$$E=\frac{\text{1}}{\text{2}}P_{F}+\frac{\text{1}}{\text{2}}{(\text{1}-P}_{D})$$ (3)

An error probability of 0.5 indicates chance-level discrimination, while an error rate of 0 indicates that the responses are different enough to support perfectly accurate discrimination.

### Coding efficiency

The size of the population of neurons used in the discrimination analysis can be varied. If it is based on the information contained in a single neuron, discrimination will be less accurate than if the information from many neurons is considered. By plotting the error probability as a function of the number of neurons included in the analysis, we can estimate how quickly the error rate decreases with increasing population size. This rate of decrease is representative of the efficiency in population coding, as it reflects the amount of information each neuron contributes. Based on this principle, we quantify a population coding efficiency by fitting an exponential function to the error probability as a function of population size:


$$F(x)=\alpha e^{-\lambda x}$$ (4)

Where λ is the efficiency rate value, and *x* is the neural population size, with higher numbers indicating a more efficient discrimination between stimuli presented from different spatial positions or orientations.

### 2D Activation Heatmaps

Two-dimensional activation heatmaps are valuable as a qualitative tool for visualizing differences in neural responses when the stimulus is presented from different spatial locations and orientations around the fish. We used a 3D mesh model of a fish’s body surface on which a population of tuberous electroreceptor locations has been placed (i.e., each dot on the fish) that was used previously in a model [40]. Briefly, the density of receptors was determined empirically for each of the faces of the 3D model, and the corresponding number of receptors was placed at random locations within the face. The use of this receptor location array was simply convenient for us and fulfilled our visualization needs. An equivalent approach of assigning response values to discrete locations would have been to choose a set of uniformly distributed locations. All receptor locations within a neuron’s receptive field boundary (determined as explained above) were assigned to that neuron, such that one receptor could belong to several neurons’ receptive field centers. Values from the neural response measures (e.g., firing rate, gain, vector strength) for a given stimulus were appended to all receptors within the neurons’ receptive field centers, and then averaged so that each receptor’s location represented a single activation value for all its represented neurons.

## Results

We investigated the spatial coding of signals for social interactions by playing conspecific stimuli from a conspecific dipole mimic (i.e., “fishpole”) while recording extracellularly from ELL pyramidal cells *in vivo*. The position of the fishpole was systematically varied (Fig 1A; see Methods), and we examined how its location or orientation influences the pyramidal cell responses. We noticed obvious qualitative differences in the neural response that ranged across the spectrum of temporal to rate aspects of the spike train. The stimulus-elicited changes in response pattern were heterogeneous, with some neurons showing pronounced changes in their mean firing rate. In contrast, other cells responded with clear differences in vector strength with little to no change in rate (Fig 1B). We could not readily identify a single aspect of the response that most clearly correlated with changes in stimulus position. We therefore used three measures in our analysis that cover the range of rate vs temporal coding: mean firing rate, gain (which reflects changes in both timing and rate of the response), and vector strength (i.e., how tightly the response is concentrated at one phase of the stimulus).

**Fig 2:**
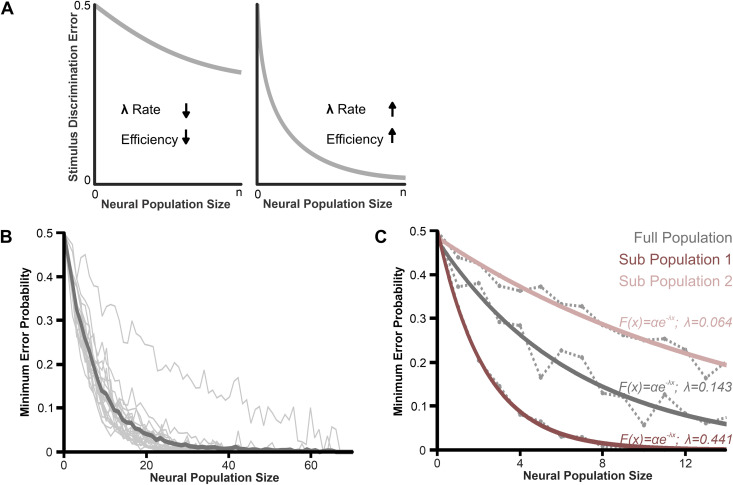
Illustration of how coding efficiency is quantified by the rate of decrease of the discrimination error as the information from more neurons is included in the analysis. **(A)** Schematic illustrating how coding efficiency is related to the rate of discrimination error. For a pairwise stimulus discrimination task, the efficiency (λ) corresponds to the slope of change in discrimination error as a function of neural population size. A slow decrease in error as the information from more neurons is pooled indicates a low coding efficiency (each neuron contributes little information). A faster decrease (right plot compared to the left) indicates a higher efficiency. **(B)** Pairwise stimulus discrimination using vector strength as a response measure, with the number of neurons used to perform the task varied (up to 70 neurons). All unique paired combinations of spatial stimuli are shown in the background (light gray), with the mean across all stimulus pairs in the foreground (dark gray). A discrimination accuracy level of 95% is obtained with fewer than 20 neurons. **(C)** Stimulus discrimination and efficiency across different neural populations. We selected two subsets of neurons (n = 14 each): one where we could see obvious differences in responses between the two stimulus locations and one where differences were not obvious. Subjectively dividing the population was done simply to create a curve with poor discrimination and one with good discrimination, as indicated by the more-or-less rapid rate of decrease of the error probability with increasing population size. By fitting an exponential function to these two curves, this panel illustrates how the parameter λ reflects the shape of the curve and thus the coding performance of the population.

We aim to compare how accurately the population of pyramidal cells encodes the spatial location of the stimulus. To do so, we compare population responses to different stimulus locations and quantify the differences in response patterns. The similarity in response pattern is based on a weighted Euclidean distance analysis that closely correlates with the amount of information the population carries about stimulus differences (i.e., location; see [29] for details). This analysis yields an estimate of the error rate in stimulus discrimination that would occur when comparing population responses. This error rate is estimated as a function of the number of neurons included in the population response. A faster decrease in error rate with increasing population size (efficiency *λ* in Fig 2) indicates that the neurons carry more spatial information. We found that all stimuli could be discriminated effectively from one another based on the patterns of population responses. When averaging across all stimulus pairs, reliable discrimination (<5% error) was achieved with a population of less than 20 neurons (see example in Fig 2B). This data shows that the spatial aspect of the conspecific stimulus is reliably encoded within the pattern of the population response. Some neurons clearly changed their response pattern for different stimulus positions, whereas others showed more subtle changes. By comparing subpopulations of neurons with clear differences for a given stimulus pair to a subpopulation with less obvious differences, we demonstrate in Fig 2C how our efficiency measure reflects the accuracy with which stimulus position is encoded in the response pattern.

ELL pyramidal cells are heterogeneous, and many differences in their response properties have been documented. Yet, it is not known whether the different subpopulations differ in their encoding of the spatial aspect of conspecific signals. To answer this question, we compare the coding accuracy across categories: ON-type vs OFF-type cells, neurons of the LS vs CLS maps, and superficial/intermediate vs deep pyramidal cells. ON and OFF-type pyramidal cells were easily distinguished based on their preference for stimulus polarity (increases vs decreases in stimulus amplitude). Location of each recorded neuron relative to the different ELL maps was estimated based on the stereotaxic position of the recording electrodes and the response properties of the neurons (see S1 Fig). In this study, we focus on the LS and CLS segments that are most relevant for processing conspecific signals [14,15]. Important differences exist between deep pyramidal cells and cells that are more superficial. We pooled together putative superficial and intermediate cells because they occupy a similar place in the circuitry of the electrosensory system, whereas deep pyramidal cells are functionally separate [6,46]. We categorized deep-type pyramidal cells based on their characteristically high spontaneous firing rate, lower coefficient of variation of their instantaneous firing rate, and their better synchronization to the EOD compared to superficial and intermediate-type pyramidal cells (S1 Fig panels D-E; [16,47–48]).

**Fig 3:**
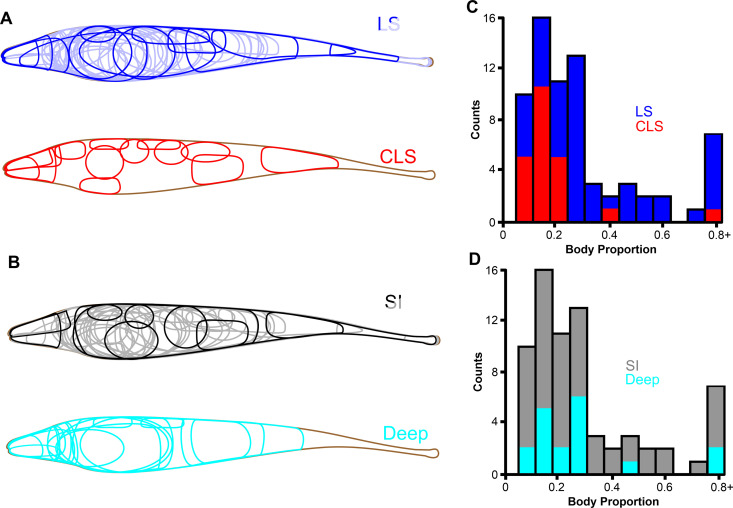
Receptive field of pyramidal cells sampled. **(A)** Boundaries of receptive field centers from recorded LS (n = 55) and CLS (n = 15) pyramidal cells on a two-dimensional outline of an *A. leptorhynchus*. Two shades of blue were used for LS receptive fields to make it easier to identify individual boundaries **(B)** Boundaries of receptive field centers from recorded deep-type (n = 18) and superficial/intermediate-type (n = 52) pyramidal cells. **(C)** Stacked histogram of receptive field sizes measured as a fraction of total body proportion for LS and CLS pyramidal cells. Mean receptive field size is larger for LS (Kruskal-Wallis, p = 0.016). As a stacked histogram, the height of the bars for each bin shows the total number of cells with a receptive field that size, and the portion with red/blue fill indicates the number of cells for each sub-population. **(D)** Stacked histogram of receptive field sizes measured as a fraction of total body proportion for deep and superficial/intermediate-type pyramidal cells. Receptive field sizes were similar between the two groups (Kruskal-Wallis, p = 0.59).

The classical receptive field of each neuron was delineated based on its response to a small local dipole that was moved across rostro-caudal and ventro-dorsal locations. The neurons we recorded had receptive fields in various positions from head to tail (Fig 3A, 3B). We note that, while most electrophysiological studies avoid sampling cells from the fish’s head because the typical experimental configuration has the fish’s head close to- or above- the water surface, we performed the experiment with the fish completely submerged in a more realistic position. We did not observe striking differences in response properties for cells of the head, despite the fact that they receive inputs from much more densely packed receptors than pyramidal cells from the trunk of the fish. Receptive field size varied from cell to cell (Fig 3B). As expected, CLS neurons had smaller receptive field sizes than LS neurons [49]. The receptive field sizes for deep-type and superficial/intermediate-type pyramidal cells were similar [6,13,46].

**Fig 4:**
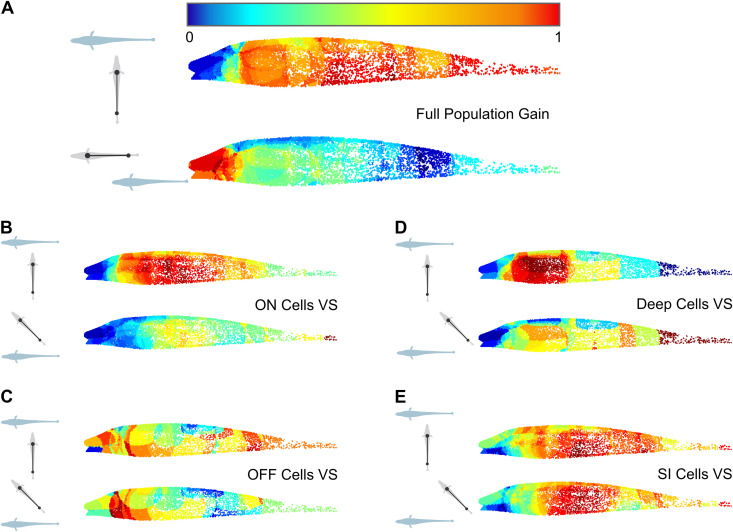
Responses to spatially realistic conspecific signals visualized as topographic heatmaps for different subsets of pyramidal cells. The conspecific stimulus is played from various relative positions and orientations. The relative position of the stimulus fishpole (grey with black dipole) and the receiver fish (light blue) is shown in the left insets (schematic is not to scale). Each colored point on the heatmap represents a putative receptor on the skin of the fish (see Methods and [40]). Its color reflects the average response strength (e.g., gain) for the neurons with receptive fields that include the point’s location. For each neuron to contribute equally to the heatmap, its responses are normalized to 1, where 1 is the strongest response of the neuron across all stimulus positions. In the five pairs of heatmaps presented here for different subpopulations of cells, we see differences across stimulus locations that vary from obvious (A, B, D) to more subtle **(C, E)**. **(A)** Heatmaps of the full population of recorded pyramidal cells (n = 70) using gain as a response measure. **(B)** Heatmaps of the ON-type pyramidal cells subpopulation (n = 46) using vector strength as response measure. **(C)** Heatmaps of the OFF-type pyramidal cells subpopulation (n = 24) using vector strength as response measure. **(D)** Heatmaps of the deep pyramidal cells subpopulation (n = 18) using vector strength as response measure. **(E)** Heatmaps of the superficial/intermediate pyramidal cells subpopulation (n = 52) using vector strength as response measure.

To visualize how ELL pyramidal cells varied in their response to stimuli from different locations, we constructed two-dimensional activation heatmaps based on one of the three response strength measurements (see Fig 1). A few examples of these heatmaps (Fig 4) were chosen to highlight a few key observations. First, as expected, stimuli from different locations lead to clear differences in the pattern of activation across the body. Also, the heatmaps highlight the heterogeneity and variability in the response pattern of pyramidal cells. Although an overall pattern of activation is visible, with receptive field facing the stimulus location being more strongly activated, pyramidal cells showed uneven patterns of activation. Undoubtedly, if more neurons were included in these representations, the gradient patterns would have been smoother. But the fact that we see these fractured gradients in regions of the body where we had particularly good sampling (mid-body) suggests that the observed heterogeneity is not a consequence of low sampling. These heatmaps also illustrate that different subpopulations of pyramidal cells may have a more obvious relationship between their response strength and the stimulus location. Specifically, we displayed responses of ON vs. OFF and deep vs. superficial/intermediate, where the ON and deep cells show clearer differences across stimulus locations.

**Fig 5:**
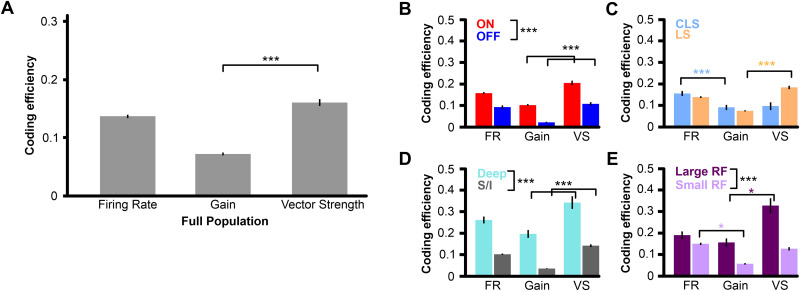
Spatial coding efficiency varies with pyramidal cell type and is dependent on the aspect of the neural response relevant for stimulus discrimination. In each panel, we show statistical comparisons between subpopulations and between the best and worst response measure (* for p < 0.01 and *** for p < 0.0001 in Tukey pairwise comparisons; asterisks beside legend labels indicate significance for the corresponding factor in the ANOVA). **(A)** Mean coding efficiency (± s.e. across stimulus pairs) of all pairwise stimulus combinations (orthogonal orientation; see Methods) using the full population (n = 70). **(B)** Mean coding efficiency (± s.e. across stimulus pairs) for populations of ON (n = 46) and OFF-type pyramidal cells (n = 24). **(C)** Mean coding efficiency (± s.e. across stimulus pairs) for populations of CLS (n = 15) and LS pyramidal cells (n = 55). **(D)** Mean coding efficiency (± s.e. across stimuli pairs) for populations of deep (n = 18) and superficial/intermediate-type pyramidal cells (n = 52). **(E)** Mean coding efficiency (± s.e. across stimuli pairs) for cells with large receptive fields (n = 33) and small receptive fields (n = 37).

Our quantitative analysis revealed that the discrimination error for specific spatial stimulus pairs varied based on the response measure used in the analysis. Overall, we found that using the mean firing rate or vector strength to quantify the response led to better spatial coding than using the gain (Fig 5A). Notably, we also investigated how the phase of the response changed in relation to the spatial stimulus. Some neurons showed noticeable shifts in response phase to a conspecific stimulus changing either orientation or location (S2 Fig). However, this measure proved less informative for population analysis when their responses were combined with either cells that also exhibited phase changes or cells that showed no phase changes in their response.

The full population was separated into distinct categories of pyramidal cell types to compare their coding efficiency. We also asked if specific pyramidal cell types would discriminate spatial stimuli more efficiently depending on the aspect of the spike train response used for discrimination. We found that ON-type pyramidal cells can discriminate the spatial stimulus more efficiently than OFF-type pyramidal cells across all three measures (Fig 5B). Similar to the full population, both ON and OFF-type pyramidal cells obtained the highest efficiency rate using the vector strength, and the lowest when using response gain. This exact finding was also observed when comparing deep-type pyramidal cells vs superficial/intermediate-type pyramidal cells (Fig 5D). However, when comparing efficiency between CLS and LS pyramidal cells, there was a flip in the measures that resulted in the highest efficiency rate. The population of CLS neurons was found to encode spatial information more efficiently using the mean firing rate. In contrast, the LS population was most efficient using vector strength (Fig 5C). The same trend is observed when comparing neurons with large receptive fields (> 0.2 body proportion) against neurons with small receptive fields (< 0.2 body proportion; Fig 5E). This might reflect the fact that LS neurons tend to have larger receptive fields than CLS neurons. Our data thus indicate that CLS and LS neurons encode spatial information with a different proportion of rate versus temporal coding, with CLS cells relying more on rate coding compared to LS cells, which encode spatial information more effectively through the timing of their responses.

**Fig 6:**
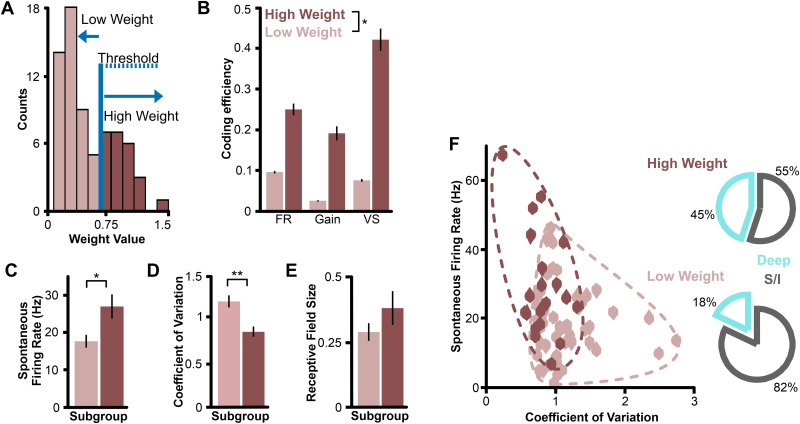
Properties of neurons with more versus less informative responses. Selected statistical comparisons are reported with asterisks (* for p < 0.01; ** for p < 0.001 in Tukey pairwise comparisons; asterisks beside legend labels indicate significance for the corresponding factor in the ANOVA). **(A)** Distribution of average weight value assigned to each neuron in the analysis that reflects the separation in their response distribution to the stimuli being compared (here averaged across stimulus pairs). A threshold was established to divide this distribution into two distinct subpopulations of neurons: low weight in pink (n = 50) and high weight in red (n = 20). We chose an arbitrary threshold corresponding to what appears as a trough in the distribution, but we do not imply that the trough or position of the threshold is significant or meaningful. **(B)** Mean coding efficiency (± s.e. across stimulus pairs; orthogonal orientations only) of the two weight groups shows the expected overall difference. **(C)** The mean spontaneous firing rate (± s.e. across neurons) for the high-weight group is higher than that of the low-weight neurons. **(D)** The mean coefficient of variation (± s.e. across neurons) of the instantaneous firing rate for the high-weight group is lower than that of the low-weight neurons. **(E)** The average receptive field size (± s.e. across neurons) for the high-weight group is higher than that of the low-weight neurons, though this difference is not statistically significant (p = 0.049). **(F)** Scatter plot of spontaneous firing rate and coefficient of variation of every neuron in each weight category. We delineated the groups with a dashed line to highlight the separation/overlap between groups. Pie charts showing the proportion of deep pyramidal cell types within each weight category (right). Note that only 18 of 70 recorded neurons are deep pyramidal cells, which represents 25.7%. Deep pyramidal cells are thus under-represented in the low-weight group and over-represented in the high-weight group.

An alternative approach to characterizing how different neurons encode spatial information is to describe the properties of neurons that carry relatively more information about stimulus location. Our decoding analysis assigns a weight to each neuron based on how different its response is to the stimulus locations being compared. This weight is based on the Kullback-Leibler divergence between response distributions, which relates to the information present in the spike trains [12,29,31,50,51]. By using the average weight assigned to a neuron across stimulus comparisons, we categorized the cells as being associated with high weights (> 0.7; Fig 6A) or low weights (< 0.7). Consequently, the cells in each group have a higher vs. lower coding efficiency (Fig 6B). This approach is complementary to our previous analysis because it allows us to determine the characteristics of cells that encode spatial information particularly well. We found that the high-weight group carried more information in their response timing (i.e., vector strength; Fig 6B), whereas the low-weight group encoded more spatial information in their mean firing rate. Interestingly, this trend parallels the differences we observed when comparing LS and CLS cells, as well as cells with large versus small receptive fields (see Fig 5). Our analysis of cell properties in each weight category also confirms the previous findings. Specifically, cells in the high-weight category had a higher average spontaneous firing rate, lower average coefficient of variation, and larger average receptive field size than neurons in the low-weight category (Fig 6C-E). We verified that putative deep pyramidal cells were more likely to be in the high-weight category (Fig 6F).

**Fig 7:**
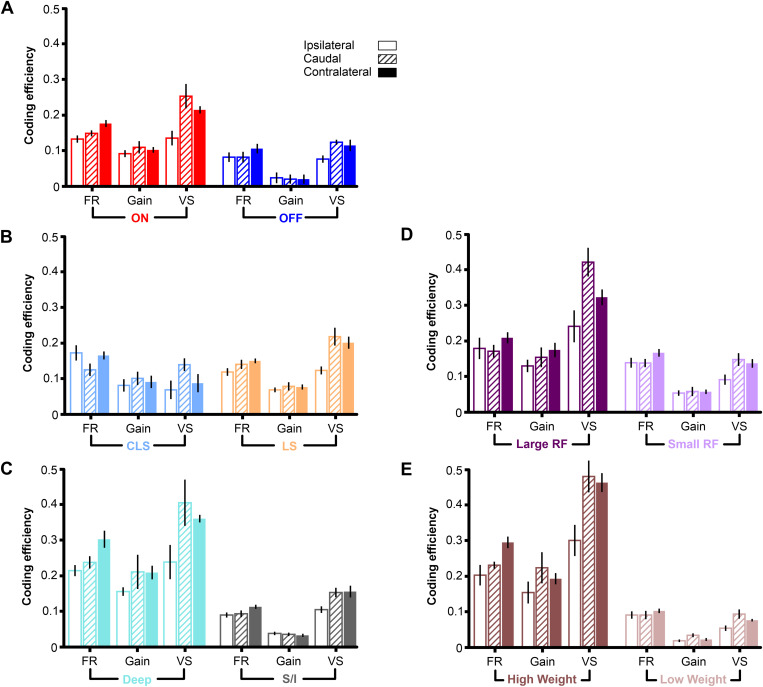
Fine and coarse spatial discrimination across cell types and response measures. Mean coding efficiency (± s.e. across stimuli pairs) for discrimination tasks where we compare: locations on the same side of the fish (ipsilateral); locations on opposite sides (contralateral); or locations on the sides compared to the caudal location. We compare different subpopulations and groups of pyramidal cells: **(A)** ON-type vs. OFF-type pyramidal cells; **(B)** CLS vs. LS, **(C)** deep-type vs. superficial/intermediate-type pyramidal cells; **(D)** large receptive field vs. small receptive field neurons, **(E)** high weight vs. low weight neurons.

The results presented in previous figures averaged the analysis of pairs of stimulus locations. We now ask if the difficulty in discriminating between spatial stimuli could be influenced by the proximity of the stimulus origins: two spatial stimuli that are close to one another may be more difficult to discriminate than two spatial stimuli that are far apart. We hypothesized that the differences we observed in spatial coding efficiency across cell types were even more apparent when considering only the more difficult discrimination tasks. Surprisingly, we found only modest differences in coding efficiency when comparing stimuli that are next to each other (“ipsilateral” to one another, Fig 7) or on opposite sides of the body (“contralateral” to one another). Overall, coding efficiency was better for coarse discrimination (contralateral: stimuli on opposite sides) than fine discrimination (ipsilateral: stimuli on the same side). The cell subtype performing best, and the response properties encoding the most information, were the same as noted in the previous analysis, and there were no qualitative differences when considering coarse versus fine discrimination tasks.

Our stimuli’s locations and intensities mimicked those of a medium-sized fish relatively close to the fish being tested. Recent findings suggest that the spatial information present in the population of receptors should be relatively accurate for stimuli at these distances [40]. It is thus probable that even when comparing our closest stimulus locations, we are not testing the lower limits of the cell’s sensitivity. Using weaker stimuli (S3 Fig), placing the stimulus source further away, or comparing locations with less separation would result in lower coding performance and potentially increase the modest differences we observe between fine and coarse discrimination tasks.

## Discussion

We investigated the spatial coding of conspecific stimuli by electrosensory neuron populations in the hindbrain. We found that this full population could discriminate accurately between all unique pairs of spatial stimuli presented. In addition to heterogeneity in anatomy and physiology, ELL pyramidal cells also vary in their functional connectivity. For example, pyramidal cells in the deep layer have no receptive field surround and do not receive feedback from either the nucleus praeminentialis or via cerebellar granular cells from the posterior eminentia granularis. In fact, deep pyramidal cells drive this feedback onto superficial and intermediate-type pyramidal cells in the ELL [4,34,52]. We therefore hypothesized that spatial coding properties for conspecific signals differ between subpopulations in the ELL. Briefly, we found that: (1) ON-type pyramidal cells displayed better spatial coding than OFF-type; (2) deep pyramidal cells outperformed superficial and intermediate-type; (3) pyramidal cells with larger receptive fields were better than those with small receptive fields; and (4) LS neurons discriminated more accurately than CLS neurons on average. By far, the clearest difference we observed in spatial coding was between the deep and superficial/intermediate pyramidal cell populations. Previous studies have shown that superficial pyramidal cells excel in temporal coding of communication signals (e.g., chirps; [14]). Interestingly, in this study, we found that superficial cells performed poorly in encoding spatial information from conspecific signals. A dichotomy between cells that encode the stimulus densely (e.g., high firing rate deep ELL cells) or sparsely (e.g., superficial ELL cells) was also observed at the next step on the electrosensory pathway, the torus semicircularis [53]. Sparse coders in the torus were specialized for encoding sensory information related to specific chirp features, whereas dense coders were more broadly responsive to electrosensory stimuli.

Electrosensory input from the ELL to the torus is topographically conserved and confined to the dorsal torus [54]. We suggest that deep ELL pyramidal cells project to dense-coder in the torus, initiating a spatial processing pathway, whereas superficial/intermediate ELL pyramidal cells might contribute predominantly to a pathway focused on temporal information. Downstream, the electrosensory pathway separates physically at the torus outputs, which project: to the optic tectum involved in spatial processing, to the nucleus electrosensorius involved in processing communication signals, and to the preglomerular nucleus that mediates connectivity with the forebrain [55–57]. Topography is conserved in the electrosensory pathway up to the optic tectum but is lost in the nucleus electrosensorius, the preglomerular nucleus, and the forebrain dorsal telencephalon (pallium). The optic tectum is therefore considered to be an important area for multisensory integration, and putatively an ultimate localization center. Taken together, our findings suggest that the separation of spatial versus identity (e.g., communication) coding begins as early as the ELL, with an early split starting between deep and superficial pyramidal cells, albeit with a possibly large overlap in function. Further studies are needed to investigate spatial coding in higher brain areas where topography remains conserved.

Several factors could explain the differences in spatial coding performance across the different sub-populations of pyramidal cells. Discrimination of stimuli location relies in our framework on differences in response strength and timing across the population. For example, the cells representing the head might fire a bit more than the cells from the tail, signaling a stimulus from a specific direction. Small differences in firing rate (or timing) across the body would need to be consistent (larger than the noise) to be a reliable source of spatial information. Deep pyramidal cells have a higher firing rate with a less variable firing pattern [58], which can lead to firing rate differences across the body that are better separated, as it will affect the mean response rate and variance. Furthermore, deep pyramidal cells do not receive feedback input. Feedback can attenuate the responses to conspecific signals and alter the variability in responses [59,60], which can -again- affect the accuracy of responses. Receptive field size can have a similar positive influence on firing rate variability and thus encoding accuracy because a larger receptive field involves pooling the responses from more receptors and thus averaging out some of the noise. Potential reasons for differences between ON and OFF cells are less obvious. OFF cells receive receptor input through an inhibitory interneuron, and their increase in firing rate reflects a disinhibition [61]. It is possible that this difference in input mechanism causes differences in response variability and sensitivity.

Our results show that certain neural subpopulations allow for more accurate discrimination when using mean firing rate, while others encode more information in their spiking pattern (phase-locking). This difference is most obvious when comparing population responses between the LS and CLS maps. Pyramidal cells in the LS map encode spatial information more accurately in their spike timing (vector strength), whereas CLS cells performed better, on average, when using mean firing rate. These differences in response properties suggest that different target populations would be more or less sensitive to each of these aspects of the pyramidal cells’ response to maximize information decoding. Several factors may contribute to the differences in coding properties that we observed here, such as receptive field parameters, ion channel composition, and the influence of feedback.

Preferences in the neural response tailored to specific stimulus features have been well documented, such as combinatorial and multiplexed neural codes [62,63]. For example, studies on human sound localization have shown how neurons that receive shared input can use asynchronous firing rates to encode the intensity of low-contrast features, while also using the precise timing of synchronous spikes to encode high-contrast features [63]. Similarly, other behavioral experiments on human sound localization have found that softer sounds can be perceived closer to the midline than louder sounds, favoring a rate-coding strategy [64]. Research on spatial navigation has shown that the time of firing can represent an animal’s location within a place field, whereas the firing rate can represent the animal’s velocity through the field [65]. Information can also be transmitted through short interspike intervals within a burst [66,67]. Thus, it is well documented that in heterogeneous neural populations, spatial information about a conspecific’s location can be represented in different aspects of the spiking response.

Our study estimates coding accuracy using a simple decoding scheme that considers the most obvious coding principles and makes no extreme assumptions. For example, our discrimination analysis uses 1-second averages of neural responses and assumes that a decoder can integrate these inputs over time. If integrating over a shorter time, because the neural system does not operate on long time scales or because the fish does not remain in a fixed location, the discrimination accuracy will decrease. Moreover, this decoding analysis might not encompass the relevant aspect of the neural response, or could underestimate coding accuracy. An alternative decoding method might implement a principal component approach to average out noise more effectively. On the other hand, our analysis could also be overestimating the coding efficiency. In our analysis, we weigh each neuron, thereby leveraging the most informative neurons over those that provide less information. It is possible that this may not be the exact computation that subpopulations of ELL pyramidal cells are performing, as our measure relates directly to the amount of information present in the system [29]. Due to these limitations, the goal of our study is not to provide absolute quantifications, but rather to serve as a means of comparing coding performance across sub-populations and identifying particularly efficient coding principles.

Our experiment considers localization in a static configuration, and movement of the stimulus location could impact circuit dynamics, particularly feedback, in a way that improves/alters spatial coding in specific subsets of cells. Superficial/intermediate would be particularly prone to this mechanism since we know that feedback can enhance their responses to small moving objects [68]. Additionally, the gathering of information can be enhanced via active sensing behaviors. Such specialized and often stereotype-patterned behaviors occur across systems and include edge detection and tracking of odor plumes in moth olfaction, and foveal sampling in the visual and electrosensory systems, to name a few [69,70]. Certain bat species have been shown to take advantage of their angle of approach with respect to the background surface to increase the signal-to-noise ratio of a prey echo during prey capture behavior. Such acoustically camouflaged prey items would normally have their weak prey echoes masked by background echoes from other objects in the natural environment [71]. High-accuracy steering towards the location of a sound source at a fixed azimuth has been documented in crickets [72]. This finding suggests that localization ability may be high when integration over time is possible. Cricket zig-zag walking and other corrective repositioning behaviors could help to re-evaluate the localization error during movement. Thus, localization during behavior should consider the motion component, and that the process from sensory to motor and back is highly dynamic. Further studies on the role of active sampling and dynamic sensorimotor adjustments are needed to better understand how the nervous system encodes the spatial aspect of signals.

In conclusion, our results provide new insights into population coding of spatially realistic conspecific signals. Our data suggest that the start of segregation of spatial processing occurs in ELL pyramidal cells. The segregation of spatial and temporal processing is a generic principle common to many sensory systems. The neural circuitry in the ELL contains several network elements that are shared across modalities, such as classical receptive field center-surround organization, topographic maps, and feedback influences that contribute to shaping the neural code. Therefore, our study provides a powerful comparative perspective to delineate general principles of sensory processing.

## Supporting information

S1 Fig Confirmation of pyramidal cell type. **(A)** Upper and lower bound coherence of ON and OFF-type pyramidal cells. Insets show spike-triggered average waveforms in response to RAM stimuli presented globally. Coherence analyses are standard and described in previous publications [31,16]. The upper-bound coherence reflects the coding accuracy, including both linearly and non-linearly encoded information, whereas lower-bound coherence is based on the linear correlation between the stimulus and the response; gray shaded areas represent ±1 s.d. across neurons. **(B)** Upper and lower bound coherence of LS ON and LS OFF-type pyramidal cells. **(C)** Upper and lower bound coherence of CLS ON and CLS OFF-type pyramidal cells. **(D)** Synchronization to the EOD between deep and superficial/intermediate-type pyramidal cells. The synchronization uses the vector strength measure (ranging from 0 to 1) in response to cycles of the EOD rather than cycles of a SAM stimulus. Deep-type pyramidal cells show higher EOD phase locking (*p* < 0.05). Vertical, black lines indicate ±1 s.e. **(E)** Scatterplot of the baseline firing rate and coefficient of variation for each neuron recorded from the full population (n = 70). Deep-type pyramidal cells are shown in blue, manually outlined for visual grouping. Superficial and intermediate-type pyramidal cells are shown in black with manually outlined grouping to better visualize clustering of cells.
(TIF)

S2 Fig Observed phase-shifted responses in ELL pyramidal cells. **(A)** Effect of stimulus orientation on a single ON-type ELL pyramidal cell. Certain neurons exhibited clear changes in phase in response to the spatially realistic conspecific stimulus. The average phase in the response is represented as θ. **(B)** 4 ON-type ELL pyramidal cells and their phase response to a stimulus with orthogonal orientation, and placed in a singular location ipsilaterally to the receptive field. Each pyramidal cell response is shown as an unfilled histogram in a color scale.
(TIF)

S3 Fig Fishpole intensity and its effects on discrimination efficiency. Discrimination efficiency of pyramidal cells (n = 23) to the spatial stimulus using two different stimulus intensities. Information to support discrimination between stimulus locations is present even for our weaker stimuli.
(TIF)
