# Supplementary figures and images for: Spatial coding of conspecifics in the electrosensory system: Early segregation of information streams
Source: PLoS One. 2026 May 12;21(5):e0348018. doi: 10.1371/journal.pone.0348018 (PMC13166950; doi:10.1371/journal.pone.0348018)

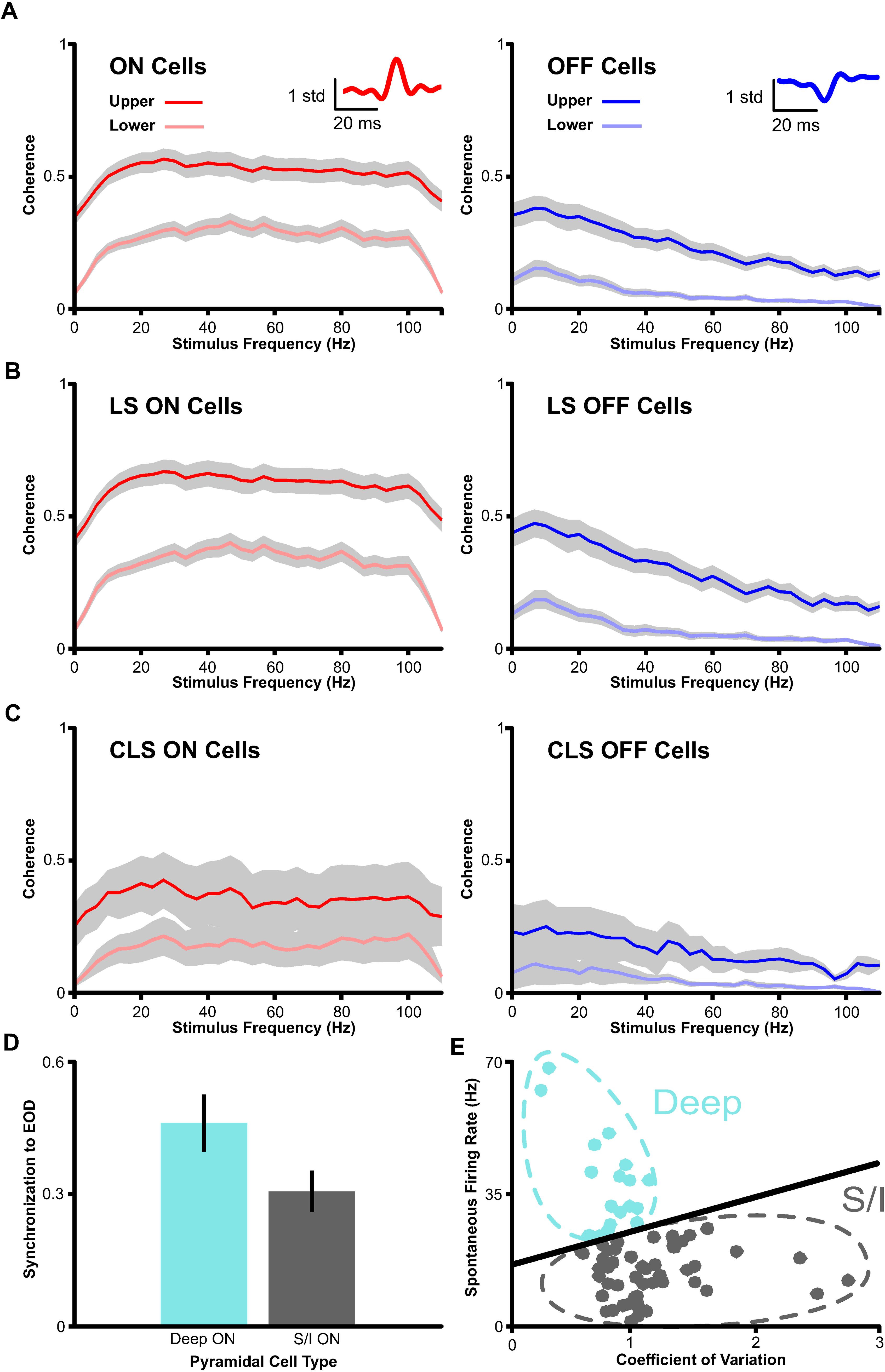

Supplement: S1 Fig — (A) Upper and lower bound coherence of ON and OFF-type pyramidal cells. Insets show spike-triggered average waveforms in response to RAM stimuli presented globally. Coherence analyses are standard and described in previous publications [31,16]. The upper-bound coherence reflects the coding accuracy, including both linearly and non-linearly encoded information, whereas lower-bound coherence is based on the linear correlation between the stimulus and the response; gray shaded areas represent ±1 s.d. across neurons. (B) Upper and lower bound coherence of LS ON and LS OFF-type pyramidal cells. (C) Upper and lower bound coherence of CLS ON and CLS OFF-type pyramidal cells. (D) Synchronization to the EOD between deep and superficial/intermediate-type pyramidal cells. The synchronization uses the vector strength measure (ranging from 0 to 1) in response to cycles of the EOD rather than cycles of a SAM stimulus. Deep-type pyramidal cells show higher EOD phase locking (p < 0.05). Vertical, black lines indicate ±1 s.e. (E) Scatterplot of the baseline firing rate and coefficient of variation for each neuron recorded from the full population (n = 70). Deep-type pyramidal cells are shown in blue, manually outlined for visual grouping. Superficial and intermediate-type pyramidal cells are shown in black with manually outlined grouping to better visualize clustering of cells. (TIF) [file pone.0348018.s001.tif]

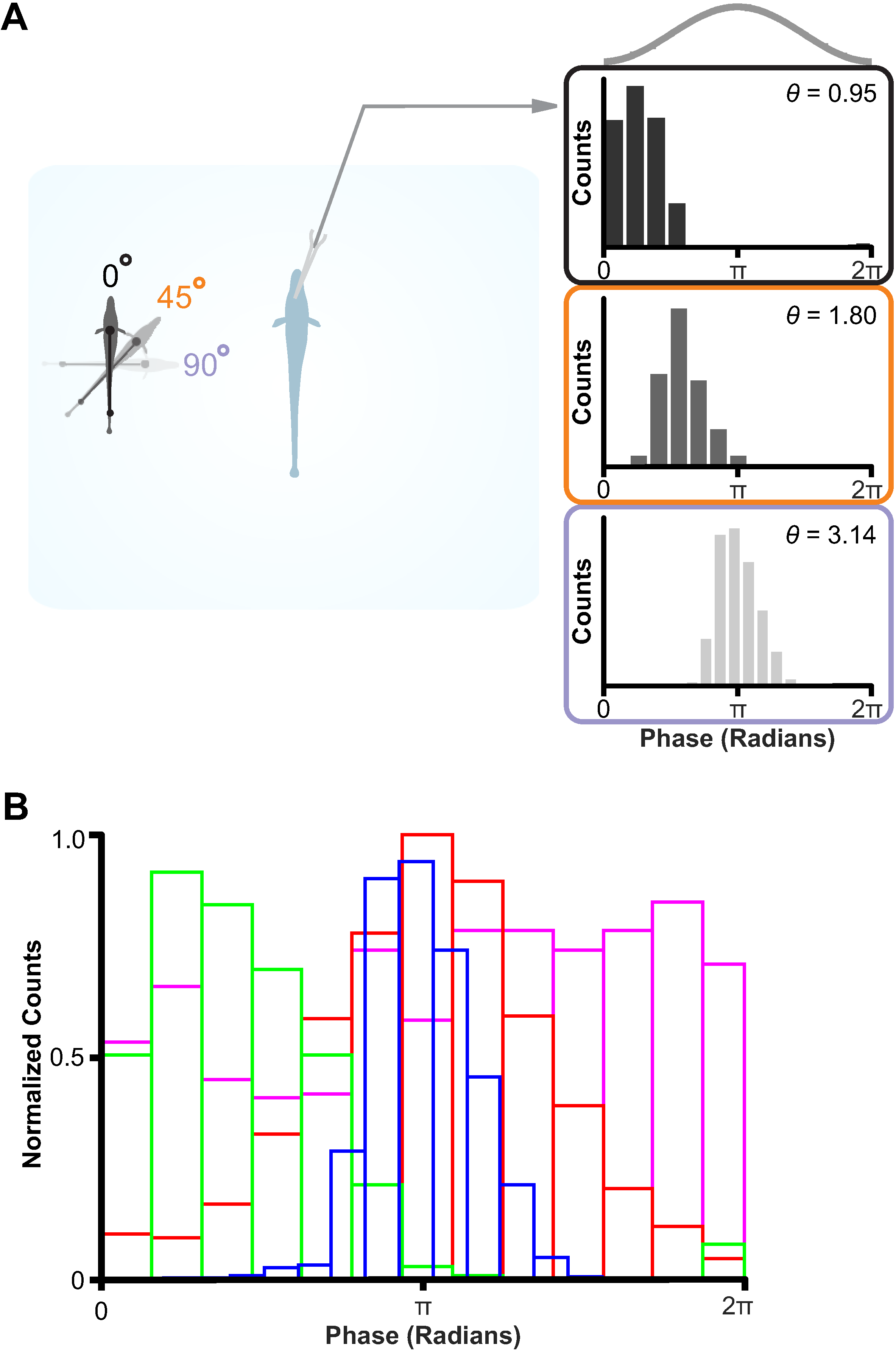

Supplement: S2 Fig — (A) Effect of stimulus orientation on a single ON-type ELL pyramidal cell. Certain neurons exhibited clear changes in phase in response to the spatially realistic conspecific stimulus. The average phase in the response is represented as θ. (B) 4 ON-type ELL pyramidal cells and their phase response to a stimulus with orthogonal orientation, and placed in a singular location ipsilaterally to the receptive field. Each pyramidal cell response is shown as an unfilled histogram in a color scale. (TIF) [file pone.0348018.s002.tif]

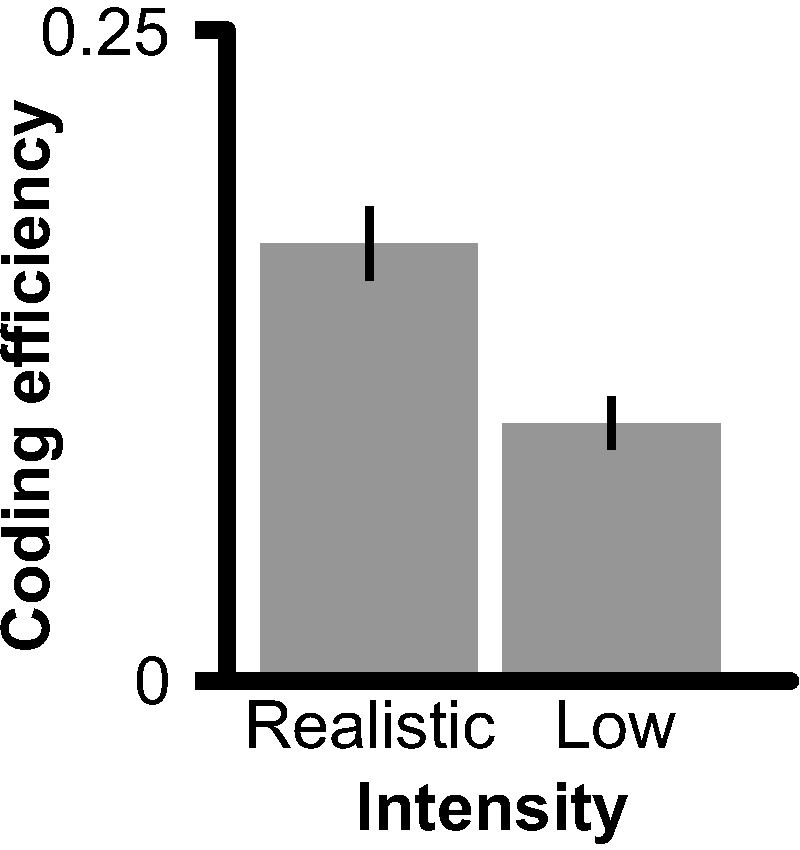

Supplement: S3 Fig — Discrimination efficiency of pyramidal cells (n = 23) to the spatial stimulus using two different stimulus intensities. Information to support discrimination between stimulus locations is present even for our weaker stimuli. (TIF) [file pone.0348018.s003.tif]
